# The application of natural language processing for the extraction of mechanistic information in toxicology

**DOI:** 10.3389/ftox.2024.1393662

**Published:** 2024-05-10

**Authors:** Marie Corradi, Thomas Luechtefeld, Alyanne M. de Haan, Raymond Pieters, Jonathan H. Freedman, Tamara Vanhaecke, Mathieu Vinken, Marc Teunis

**Affiliations:** ^1^ Innovative Testing in Life Sciences and Chemistry, Utrecht University of Applied Sciences, Utrecht, Netherlands; ^2^ ToxTrack, Bethesda, MD, United States; ^3^ Environmental Health and Engineering, Johns Hopkins Bloomberg School of Public Health, Baltimore, MD, United States; ^4^ Institute for Risk Assessment Sciences, Utrecht University, Utrecht, Netherlands; ^5^ Gillings School of Global Public Health, University of North Carolina at Chapel Hill, Chapel Hill, NC, United States; ^6^ Department of Pharmaceutical and Pharmacological Sciences, Vrije Universiteit Brussel-Belgium, Brussels, Belgium

**Keywords:** natural language processing, toxicology, adverse outcome pathway, risk assessment, machine learning, open science

## Abstract

To study the ways in which compounds can induce adverse effects, toxicologists have been constructing Adverse Outcome Pathways (AOPs). An AOP can be considered as a pragmatic tool to capture and visualize mechanisms underlying different types of toxicity inflicted by any kind of stressor, and describes the interactions between key entities that lead to the adverse outcome on multiple biological levels of organization. The construction or optimization of an AOP is a labor intensive process, which currently depends on the manual search, collection, reviewing and synthesis of available scientific literature. This process could however be largely facilitated using Natural Language Processing (NLP) to extract information contained in scientific literature in a systematic, objective, and rapid manner that would lead to greater accuracy and reproducibility. This would support researchers to invest their expertise in the substantive assessment of the AOPs by replacing the time spent on evidence gathering by a critical review of the data extracted by NLP. As case examples, we selected two frequent adversities observed in the liver: namely, cholestasis and steatosis denoting accumulation of bile and lipid, respectively. We used deep learning language models to recognize entities of interest in text and establish causal relationships between them. We demonstrate how an NLP pipeline combining Named Entity Recognition and a simple rules-based relationship extraction model helps screen compounds related to liver adversities in the literature, but also extract mechanistic information for how such adversities develop, from the molecular to the organismal level. Finally, we provide some perspectives opened by the recent progress in Large Language Models and how these could be used in the future. We propose this work brings two main contributions: 1) a proof-of-concept that NLP can support the extraction of information from text for modern toxicology and 2) a template open-source model for recognition of toxicological entities and extraction of their relationships. All resources are openly accessible via GitHub (https://github.com/ontox-project/en-tox).

## Introduction

The toxicity of a stressor (e.g., compound or environmental stressor such as radiation) is defined by its potential for causing harmful effects on an individual, a population, or ecosystem (ecotoxicity). In this report, we will focus on the organism level. Traditionally, the toxic potential of a stressor is evaluated by performing tests on animals, in particular mammals, and assuming the obtained results can be extrapolated to humans. This approach is being increasingly questioned for two main reasons. First, the ethicality of sacrificing millions of animals each year (Fernandes and Pedroso, 2017). Second, it is becoming increasingly clear that the effects on animals often translate poorly to humans (Van Norman, 2019). Toxicological effects are therefore preferentially studied in a mechanistic way, where toxicologists try to understand the cascade of biological events leading to a given adverse outcome (Hartung et al., 2012), and taking into account the specificities of human physiology compared to other organisms. This opens the door to the development of approaches at lower levels of biological organization, such as toxicogenomics (Waters and Fostel, 2004), and to start viewing toxicological assessment as probabilistic rather than deterministic, i.e., toxic vs. non-toxic (Bus, 2017; Maertens et al., 2022).

Such a mechanistic approach is taken in the development of adverse outcome pathways (AOPs). AOPs are frameworks that link an initial perturbation, the molecular initiating event (MIE) to an organ or organism toxicity manifestation or adverse outcome (AO) through a series of steps at different levels of biological organization, the key events (KE) (Ankley et al., 2010). Consecutive KEs are linked by key event relationships (KER) which represent a causal relationship between two KEs. As such, AOPs provide a way to organize mechanistic information leading to pathologies in humans, and to guide the development of new approach methodologies to evaluate toxicity using human-based *in vitro* tests and/or *in silico* models. Most existing AOPs are gathered in the AOPWiki (https://aopwiki.org/). Building or expanding them however relies on gathering, reviewing and synthesizing a wealth of existing knowledge, very often in the form of loosely structured text such as scientific literature or regulatory reports. This process is time-consuming and tedious, and it could be argued a better use can be made from the time of experts.

We proposed recently that developments in the field of Natural Language Processing (NLP) could allow toxicologists to screen more efficiently through literature (Corradi et al., 2022). NLP is a field of machine learning that focuses on the analysis of text and the extraction of information from it. Interestingly, models have been developed to extract biomedically relevant entities from text (Neumann et al., 2019). However, extracting relationships between these entities, which are essential for the mechanistic approach in AOPs, proves to be difficult. While promising approaches have been developed in the field of Relationship Extraction (RelEx), some even to support AOP development itself, they tend to be based on co-occurrences of terms (Zaslavsky et al., 2021; Jaylet et al., 2023), focus on compound-disease relationships (Katritsis et al., 2022), or rely on generative models which are “black boxes” and require a lot of computing power (Huguet Cabot and Navigli, 2021). Here, we used a comparatively simpler grammatical rules-based approach that integrates some linguistic information. It allows us to manually examine the relationships extracted as the results are fully retraceable to the original scientific abstracts that were used as a source.

While AOPs represent general biological pathways leading to adverse outcomes, and are hence designed to be stressor-agnostic (Villeneuve et al., 2014), in practice their MIEs tend to be triggered by prototypical compounds. We conducted a case study on a collection of compounds and their expected associated adverse outcome(s) curated by ASPIS consortium members (https://aspis-cluster.eu/), and more specifically those included in the ONTOX project (Vinken et al., 2021). The ASPIS cluster is a collaboration of three European projects (ONTOX, PrecisionTox, RISK-HUNT3R) with a common goal towards animal-free chemical risk assessment. ONTOX in particular is looking to develop a strategy to predict toxicity without the use of animals driven by mode-of-action ontologies and artificial intelligence. For that reason ONTOX is looking at multiple case studies (adverse outcomes in diverse organs). We extracted literature associated with the entire collection of compounds from PubMed, and analyzed it using a custom-made NLP pipeline. We then verified whether we were able to extract relevant information regarding adverse outcomes and the mechanisms that led to them.

Manifestations of drug or compound adversity are frequently observed in the liver. The enzymatic processes in the liver, aimed at detoxifying compounds from the bloodstream, can contribute to adversities observed in this organ. Two adversities that are generally observed during post-marketing surveillance are cholestasis and steatosis. These adverse outcomes are therefore of particular interest for the ONTOX project. Cholestasis, the accumulation of bile, is a major drug-induced adverse outcome and can be responsible for severe liver damage and increased morbidity. Steatosis is the accumulation of small (microvesicular steatosis) or larger (macrovesicular steatosis) fat droplets. We will focus in this study on these two liver adverse outcomes.

## Methods

### Literature retrieval

We started from a curated list of 813 compounds of interest for the three projects composing ASPIS (https://aspis-cluster.eu/). This list groups different types of compounds associated with one or multiple adverse outcomes of interest to one of the projects from the cluster, including cholestasis and steatosis. In the case of cholestasis and steatosis, these were selected by based on literature. For each compound, we programmatically retrieved a maximum of the first 100 abstracts found by querying PubMed for: compound name AND toxic* AND (human OR Animals, Laboratory OR Disease Models, Animal). For that purpose we used the Python packages metapub (v0.5.5) for querying and biopython (v1.79) for text retrieval. The query was restricted to 100 abstracts because of computational resources and because we intended to carry out a proof of concept. No minimum number of abstracts per compound was defined, however we analyzed how many were found for each compound (see results section). Duplicate abstracts were removed.

### Preprocessing

All steps were conducted using the open-source Python package spaCy v3.0.8 (Honnibal and Montani, 2017). Basic pre-processing such as sentencizing (breaking an abstract into its individual sentences), tokenizing (breaking the sentences into words) and semantic parsing (identifying the grammatical roles of each token) was performed to support our Named Entity Recognition (NER) and Relationship Extraction modules.

### Named Entity Recognition

Named Entity Recognition refers to the identification of spans of words in the sentences that refer to concepts of interest. In this case, we extracted the entities COMPOUND, referring to a chemical compound or substance, and PHENOTYPE, referring to a biological event. For this we used a machine learning model. Specifically, the NER model was trained using scispaCy en-core-sci-lg (Neumann et al., 2019) as a starting point, which allowed for a vocabulary (word vectors) and grammar trained on scientific literature. It was re-trained to recognize mentions of toxicological concepts, including compounds and phenotypes in scientific texts. This included both PubMed articles and ECHA reports. The training corpus was annotated by members of the project team with a background in biology, with a manual comprising a detailed description of the entities available to annotators. In particular, we define a phenotype as a biological effect at any level: molecular, cellular, organ or organism. This NER model was trained on a DART (developmental and reproductive toxicity)—oriented corpus (Bhalla et al., 2023), but generalizes to most types of organism-level phenotypes (see results). Cross-validation analysis on the training corpus showed an F1 score of 56% on phenotypes and 88% on compounds. As a consequence, we expected the model to identify compounds better than phenotypes.

### Relationship extraction

**FIGURE 1:**
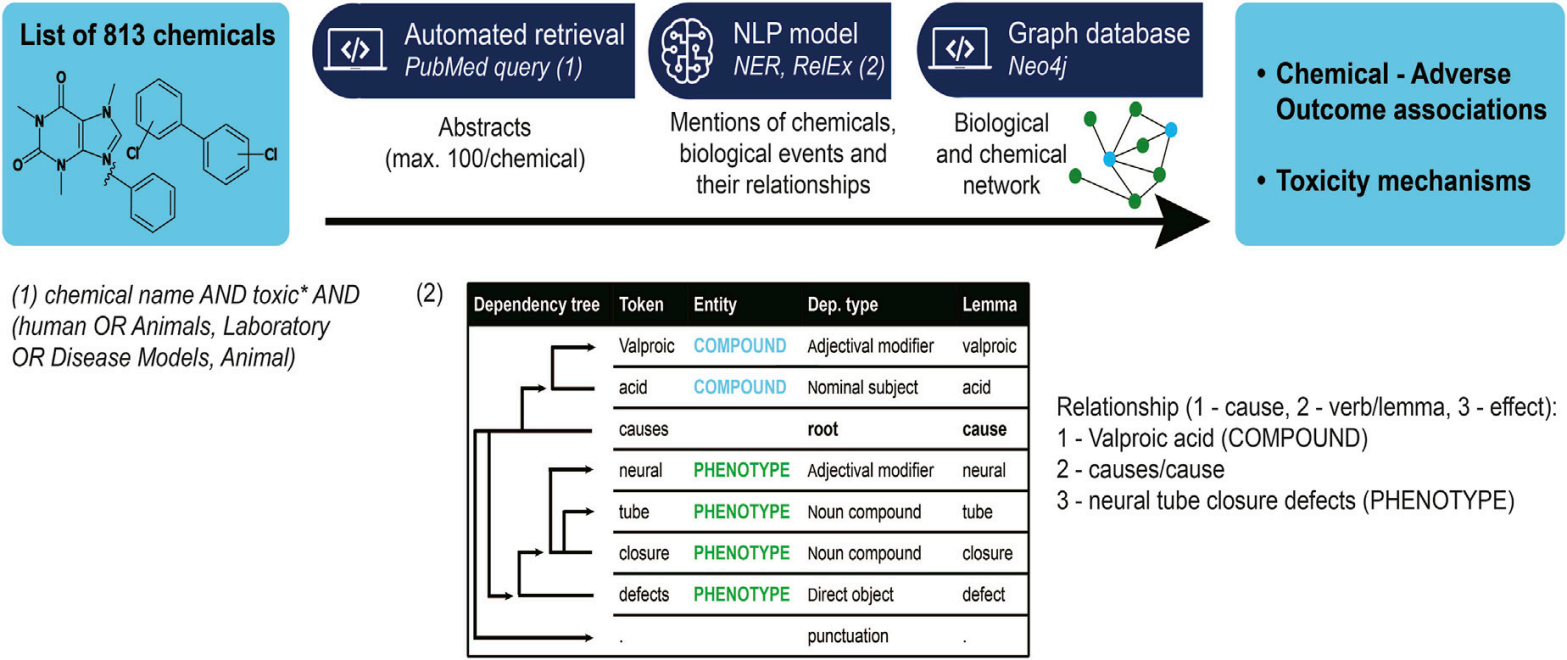
Information extraction workflow, from a list of compounds to the abstracts associated with them, to relationships between toxicological-relevant entities in these abstracts. Insert (1) shows the PubMed query used to find articles associated with toxicity for a certain compound. We restricted ourselves to human or animal-associated results as they are still the gold standard for now. Insert (2) details the relationship extraction mechanism: a relationship between two given entities is identified if a (predetermined) causal verb is a common ancestor in the semantic tree of the phrase containing both entities.

We established a semantic rules-based model for causal relationship extraction between identified entities, using spaCy’s Dependency Matcher. Two entities were considered causally related if they had a common causal verb ancestor in their semantic tree. The list of causal verbs contained the following terms: “increase”, “produce”, “cause”, “induce”, “generate”, “effect”, “provoke”, “arouse”, “elicit”, “lead”, “trigger”, “derive”, “associate”, “relate”, “link”, “stem”, “originate”, “lead”, “bring”, “result”, “inhibit”, “elevate”, “diminish”. More precisely, we verified whether the lemma of the verb, in other terms its base form, was a common ancestor in the semantic tree. Using the lemma prevents the conjugation of the verb to affect the results. We extracted relationships between identified phenotypes, possibly at different levels of biological organization, as well as between compounds and phenotypes (Figure 1). For further information about the model please review the readme on the GitHub repository (https://github.com/ontox-project/en-tox).

### Neo4j network

The resulting entities and their relationships were then organized into a Neo4j database (version 4.4.5), where each node was an entity (phenotype/compound) and each edge an identified relationship between two entities. The edges also referenced the article the relationship was extracted from. We explored the graph by querying it for specific adverse outcomes of interest. The queries can be found in the GitHub repository (https://github.com/ontox-project/en-tox). We verified whether we were able to find compounds associated with the liver adverse outcomes of interest, by querying for cholestasis or steatosis and finding the first level connections in the network.

## Results

### Automatic literature retrieval

Table 1 summarizes the number of articles found per compound in the ASPIS list. We note that for a significant amount of compounds, about 34%, no abstract was retrieved, which means we most likely will not be able to extract any information about them (there is a small chance there will be information about them in an abstract designated to another compound). For compounds associated with a liver adverse outcome (cholestasis or steatosis) in the ASPIS curated list (see methods), this number is close to 20%. For 35% of compounds (50% for cholestasis/steatosis compounds) we reached the maximum number of abstracts we could extract, which means there is most likely more literature available.

**TABLE 1:** Number of abstracts retrieved per compound.

Number of abstracts retrieved	Number of compounds (complete list/Liver AO-related)
0	278/14
0 < *n* < 10	68/5
10 ≤ *n* < 100	185/22
100	282/40

The first number refers to the number of compounds in the complete list, the second number to the compounds associated with a liver adverse outcome (cholestasis or steatosis) only. For example, 278 compounds in the ASPIS list had no abstract associated to them, 14 of these compounds were cholestasis or steatosis-related.

We further investigate the results in two different directions. First, we verified whether we were able to find compounds associated with a given liver adverse outcome, cholestasis or steatosis. Second, we evaluated how much mechanistic information we were able to retrieve in the process. For that purpose, we query sub-parts of the graph obtained in Neo4j.

### NLP supports compound selection for liver adverse outcomes

As discussed, we queried our Neo4j database for “cholestasis” (respectively “steatosis”) and all its neighbor nodes labeled as “COMPOUND”.

### Cholestasis

**FIGURE 2:**
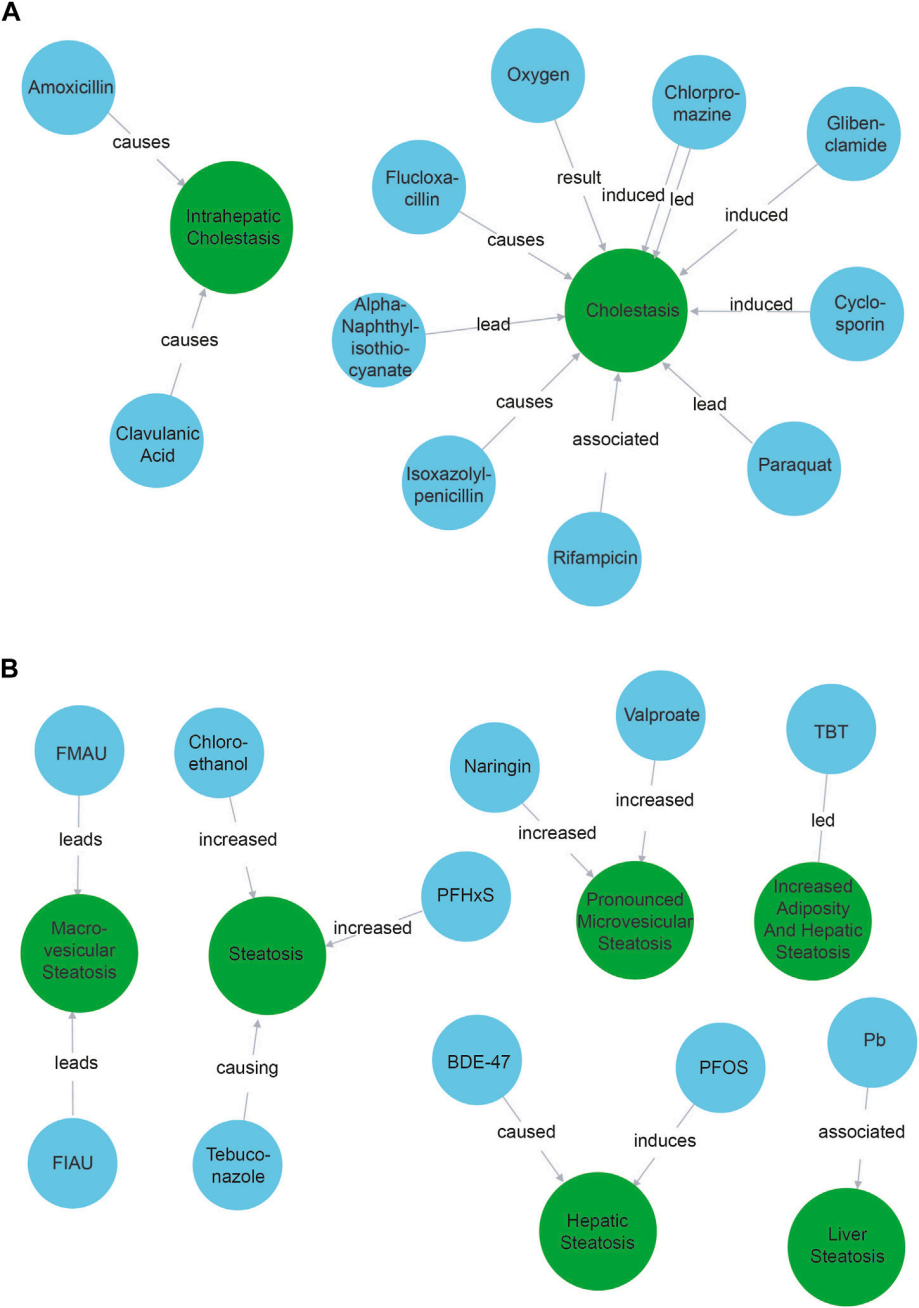
Compounds associated with cholestasis **(A)** and steatosis **(B)**. Blue nodes refer to compounds while green nodes refer to phenotypes. The causal verbs connecting the entities are depicted on the arrow between them.

We observed that all 10 compound nodes found with the cholestasis query were indeed identified as positive controls known to trigger a cholestasic adverse outcome (Figure 2A). The only notable exception was the node “oxygen”. This refers to the production of reactive oxygen species (Yu et al., 2017), e.g., event 249 in the AOPWiki, and is a known artifact of our model that tends to identify compounds better than phenotypes.

### Steatosis

The results when querying for “steatosis” were a bit more nuanced (Figure 2B). We found 11 compound nodes. Two of the compounds were associated with steatosis as described in the ASPIS list: TBT (tributyltin) and Valproate, while another two (Pb—lead and PFOS - Perfluorooctanesulfonic acid) were related to cholestasis. Five compounds were not explicitly associated with steatosis, but were indeed associated upon verifying literature manually: BDE-47 (Wang et al., 2018), PFHxS (Perfluorohexane sulfonic acid) (Jin et al., 2020), Tebuconazole (Ku et al., 2021), FIAU (fialuridine) (Cui et al., 1995) and chloroethanol (Anders et al., 2016). FMAU is not *per se* a compound but a metabolite of fialuridine, which is linked to steatosis (Cui et al., 1995). Interestingly, chloroethanol was found in the NLP pipeline, but is not (yet) included in the ASPIS list. Finally, naringin was found, which is described as protective for steatosis/liver damage (Guan et al., 2023). Our NLP pipeline could thus extract information from the abstracts about 10 known steatosis related compounds, only 2 of which were explicitly expected. We thus identified 8 possible candidates to be considered in AOP development. In addition, we retraced the result about naringin back to the original scientific abstract which showed that naringin was mentioned in the same sentence as valproic acid and wrongly lumped together as associated with cholestasis. This indicates that when using machine learning to identify possible KEs, data lineage and using explainable AI methods are important.

### Missing compounds

We did not find 53 of the 81 compounds (65%) that could be expected based on the ASPIS list. For 14 of those (17%) 0 abstracts were found. Manually querying the other missing compounds and their direct neighbors in the Neo4j network showed that in fact 16 of them (20%) were found to be related to a more general form of liver damage such as “hepatotoxicity” or “liver injury.” We expect that more specific information would be contained in the full text papers. Overall, the pipeline thus retrieved 44 out of 81 compounds, which is 54%.

We observed that complicated compound names (e.g., “5-Amino-6-chloro-o-cresol”) are not always used in abstracts of their papers, and therefore are not extracted by the NER pipeline. Similarly, very general compound names (e.g., “Basic brown 17”) tended to be not recognized as compounds. Also, compound names that include other compounds (e.g., “valproic acid sodium salt” containing “valproic acid”) tended to get overlooked.

Overall, these results show that our approach can contribute to identifying compounds associated with adverse outcomes of interest, maybe including different stressors than the ones toxicology experts traditionally consider. We do remark that entities could be better unified: “steatosis” triggered five separate entities that could be merged into one or two. For example, “liver steatosis” and “hepatic steatosis” are virtually synonyms. This highlights the need for disambiguation and normalization of entities, possibly by linking them to existing identifiers.

### NLP pipeline extracts mechanistic information at a high level of biological organization

From AOPWiki, we extracted all events (MIE, KE and AO) pertaining to all AOPs related to our liver adverse outcomes of interest, steatosis and cholestasis. The corresponding AOPs are listed in Table 2. AOPs 59 and 421 both comprise only one key event, respectively a MIE and a KE. We excluded them from further analysis.

**TABLE 2:** Current available AOPs in AOPWiki related to either cholestasis or steatosis.

AOP	AO	Title
27	Cholestasis	Cholestatic Liver Injury induced by Inhibition of the Bile Salt Export Pump (ABCB11)
421	Cholestasis	PPARG activation leading to intrahepatic cholestasis
34	Steatosis	LXR Activation to Liver Steatosis
36	Steatosis	Peroxisomal Fatty Acid Beta-Oxidation Inhibition Leading to Steatosis
57	Steatosis	AhR activation to steatosis
58	Steatosis	NR1I3 suppression to steatosis
59	Steatosis	HNF4A suppression to steatosis
60	Steatosis	PXR activation to steatosis
61	Steatosis	NRF2/FXR to steatosis
62	Steatosis	AKT2 activation to steatosis
232	Steatosis	NFE2/Nrf2 repression to steatosis
318	Steatosis	GR activation leading to hepatic steatosis

Note that there are more AOPs available for steatosis. PPARG, Peroxisome Proliferator-Activated Receptor Gamma; LXR, Liver X Receptor; AhR, aryl hydrocarbon receptor; NR1I3, Nuclear Receptor Subfamily 1 Group I Member 3; HNF4A, Hepatocyte Nuclear Factor-4 alpha; PXR, Pregnane X Receptor; NRF2/Nrf2, Nuclear factor erythroid 2-related factor 2; FXR, Farnesoid X Receptor; AKT2, AKT Serine/Threonine Kinase 2; NFE2, nuclear factor, Erythroid 2; GR, glucocorticoid receptor.

**FIGURE 3:**
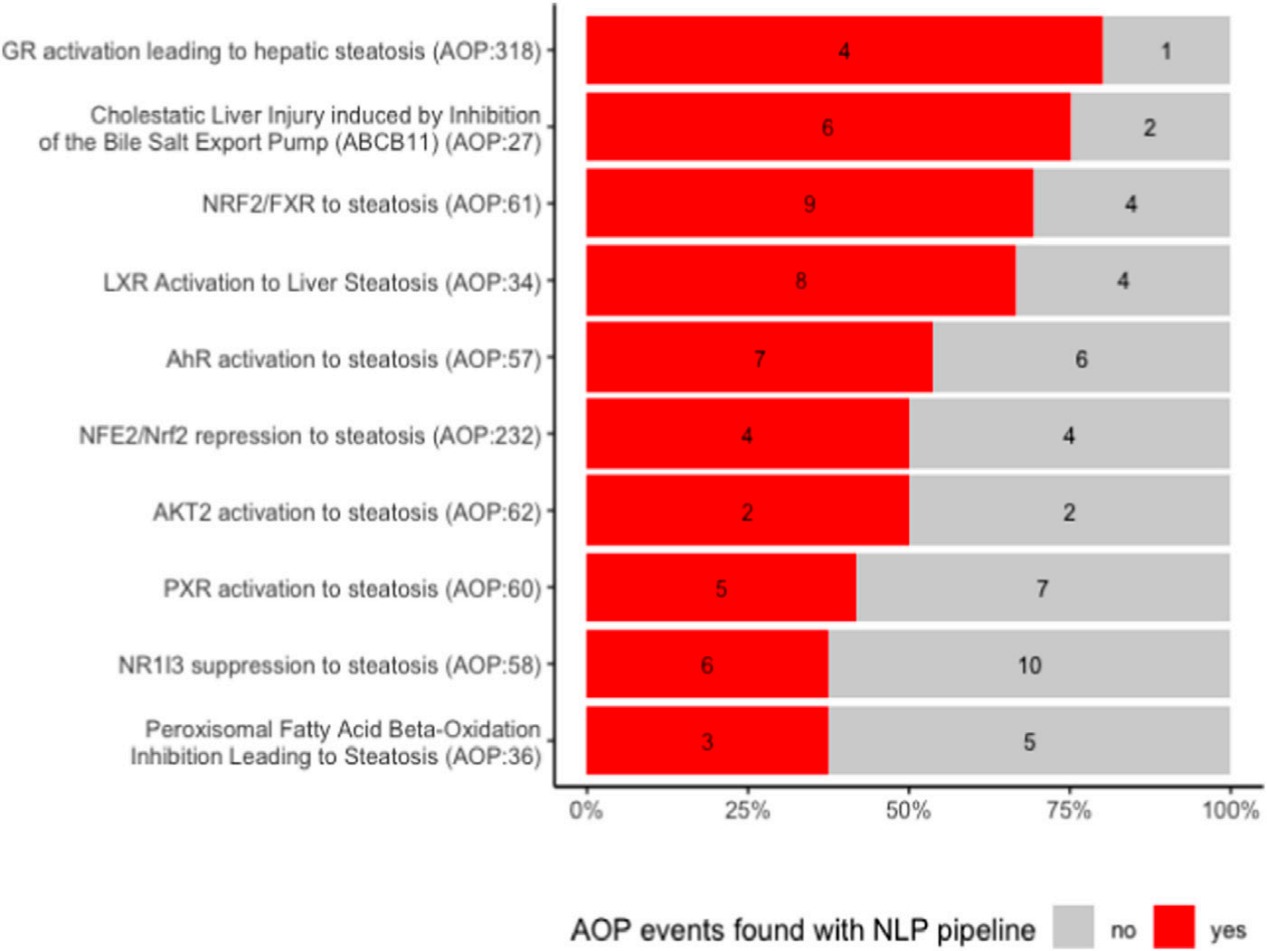
Number of key events found with the NLP pipeline and manual curation.

We first examined manually how many of the events described in the AOPs we found back with our NLP pipeline. This is depicted in Figure 3.

**FIGURE 4:**
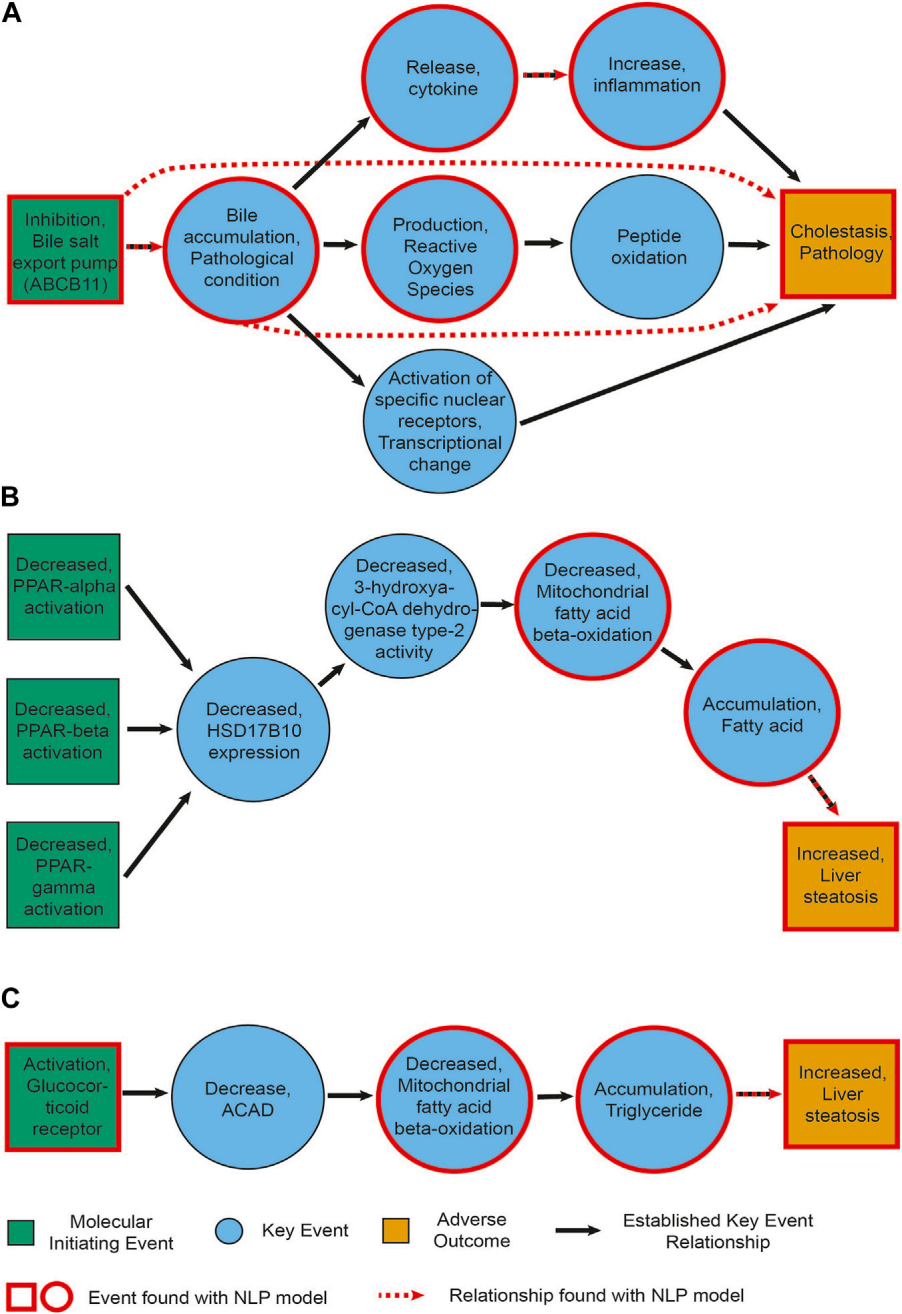
Information extracted for AOPs. **(A)** AOP27 [Cholestasis and Cholestatic Liver Injury induced by Inhibition of the Bile Salt Export Pump (ABCB11)]. **(B)** AOP36 (Peroxisomal Fatty Acid Beta-Oxidation Inhibition Leading to Steatosis). **(C)** AOP318 (GR activation leading to hepatic steatosis). Green and yellow boxes represent MIEs and AOs, respectively. Blue circles show the key events between them. Black arrows depict established KERs. Events circled in red were also found by our NLP pipeline upon manual curation. Red dashed arrows represent relationships extracted by the NLP pipeline (including non-adjacent ones, e.g., relationships between an MIE and an AO).

For each AOP, we retrieved between 40% and 80% of events. When looking more closely at what type of events we were able to extract, we observed that all events that were not identified are related to a higher level of biological detail. They are mostly associated to a specific gene or protein, e.g., a change in its activation or expression, and often belong to the MIE type. For example, none of the activations of peroxisome proliferator activated receptors (PPAR-alpha, beta and gamma), the MIEs of AOP 36, were recognized by our model (see also Figure 4B). This can be partially explained by the fact that our NER pipeline recognizes higher (biological) level phenotypes better. It might be remedied by re-training our model on a corpus containing more mentions of molecular or cellular level phenotypes.

Figure 4 depicts the events and KER extracted with our NLP pipeline for cholestasis (AOP 27) and steatosis (AOP 36 and 318, where we found the lowest and highest percentage of events, respectively). Each panel is a representation of an established AOP adapted from AOPWiki and depicts in green and yellow boxes the MIE(s)/AO, respectively, in blue circles the key events between them, and in black arrows the KERs. Events circled in red were also found by our NLP pipeline upon manual curation. Red dashed arrows represent relationships extracted by the NLP pipeline as well. While we found a number of key events, we did not always directly extract the links between them (i.e., the KERs). This seems to point to a necessary refinement of our relationship extraction module, in particular again between molecular-level phenotypes. Interestingly, in two instances we did find a link directly between an event and the adverse outcome this event would trigger after a cascade of other events, such as between bile accumulation and cholestasis (Figure 4A).

## Discussion

We showed that NLP can help extract mechanistic information from text, as well as provide a screening of stressors and their associated adverse outcomes from the literature, possibly unearthing less commonly used stressors for testing by toxicologists. We provide the model used for entities and relationship recognition in a reproducible container. We foresee the use of NLP allowing the AOP framework to exist more as a living, dynamic systematic review, where new information about MIEs or KEs could be integrated (almost) as soon as it is published.

However, our results show that several steps are still needed. Firstly, the automation of disambiguation and unification of biological events and compounds as well as their linking to semantic ontology terms would help make the extracted data more interoperable and reusable. For example, by connecting a phenotype entity directly to the corresponding KE in AOPWiki, this technique would allow the researcher to record evidence pertaining to KERs easily. Only if an event does not exist, would a new identifier be created, perhaps in first linking it to other ontologies such as Gene Ontology, UMLS, or Mammalian/Human Phenotype Ontology. We noticed with this study that a number of KEs seem to be very similar, only having slightly different phrasings, for example, events 459 (“Increased, Liver Steatosis”) and 1,418 (“Increased, steatosis”), or 115 (“Increase, FA Influx”) and 465 (“Increased, FA Influx”). This might be a consequence of manually building AOPs, as it can be time-consuming to verify every key event that already exists. In that regard, NLP could help AOP developers not to duplicate existing information.

Secondly, the relationship extraction model can be improved. While our NLP pipeline can extract relational information from abstracts, it clearly did not find all the information that could have been expected. We suggest training the model on lower level information. In addition, linguistically complex (sub)sentences are difficult to identify.

Recent developments in the field of Large Language Models (LLMs) could be of use, as some promising results have been achieved in the field of relationship extraction, and in generally finding information in, e.g., scientific documents (retrieval augmented generation) (Bran et al., 2023). We remain cautious though as the large generative models have been known to “hallucinate”, giving factually wrong answers to queries (Ji et al., 2023). The model should then be restricted to find answers in the given text(s) as our model is currently doing. A possible tool to do so would be a similar approach to Lála et al. (2023), which answers questions by looking in documents and finding the most relevant passages, thanks to similarity in embeddings between the question and the text in documents. LLMs are also computationally expensive, and therefore tend to be run in the cloud. This has by extension a larger environmental impact (Monserrate, 2022). An approach using them should hence be preceded by a cost/benefit evaluation.

We envision a model where a relationship is not expressed as a binary event (existing/non existing), but where a probability of existence is associated. We could also record when an event is explicitly NOT happening, or counteracted in some way. In addition, the incorporation of contextual and/or quantifiable events, such as the administration dosage and route of a compound, the type of test performed (*in vitro/in vivo/in silico*), relevant biological information (sex, organism), or change in the amount of an event, should also be incorporated into the model, contributing to this probabilistic prediction. The extraction of additional context could also support the development of quantitative AOPs and/or Physiologically-Based Kinetic (PBK) models.

We furthermore expect we could have been able to reconstruct better the AOPs had we not limited ourselves in the number of articles we analyzed. Here, the amount of evidence will be a key factor in determining the solidity of the mechanism. A possible follow-up would be to apply our NLP pipeline to the entirety of PubMed. We could also consider applying it to the entirety of PMC (PubMed Central), to analyze full text rather than only abstracts. However, this would come with its own set of challenges. First, our model was trained on abstracts, and is not guaranteed to generalize well to full text or Results sections, as these might be written in a different style than abstracts (Westergaard et al., 2018). Secondly, we would be limited and biased by the fact that we would only be able to analyze Open Access articles, as for articles behind paywalls only the abstract is available. Third, we suspect the computing time necessary to analyze all PubMed/PMC is considerable (as of August 2023, there are over 36 million papers on PubMed), and therefore not necessarily in line with an environmentally mindful approach. We would therefore need to implement some screening procedure beforehand. We could, for example, use existing resources linking compounds and PubMed articles such as Chembl (Mendez et al., 2019) or ctdbase (Davis et al., 2023), to query articles related to prototypical stressors.

It would be wiser at this stage to keep a human in the loop to verify the quality of the information extracted, because we observed that one compound was retrieved that is actually known to be protective for liver damage. This NLP model could be used as an aid for AOP building, where the toxicologist can spend their valuable time controlling and assembling the information automatically extracted from literature, instead of reading dozens of articles.

In this report we demonstrate how the information generated via our NLP pipeline can aid in the creation of AOPs. The application of this information, including physical-chemical properties, cross-species toxicity, exposome, pathology, etc., is limited only by the needs of the user. For example, our NLP pipeline can be applied to larger-scale extraction of chemical effects to supplement QSAR, read-across approaches, as well as contributing to next-generation risk assessment frameworks. By extracting adverse effects associated with various chemicals from the existing literature, we can supplement databases with structural, physico-chemical and biological properties to facilitate the development of models predicting the toxicity of (new) chemicals with a similar structure or properties. It is likely that the information collected from the literature using the NLP pipeline can be applied to and impact virtually all aspects of toxicology and risk assessment.

## Data Availability

The datasets presented in this study can be found in online repositories. The names of the repository/repositories and accession number(s) can be found below: https://github.com/ontox-project/en-tox/tree/main/article/data.

## References

[B1] Anders L. C. Yeo H. Kaelin B. R. Lang A. L. Bushau A. M. Douglas A. N. (2016). Role of dietary fatty acids in liver injury caused by vinyl chloride metabolites in mice. Toxicol. Appl. Pharmacol. 311, 34–41. 10.1016/j.taap.2016.09.026 27693805 PMC5079761

[B2] Ankley G. T. Bennett R. S. Erickson R. J. Hoff D. J. Hornung M. W. Johnson R. D. (2010). Adverse outcome pathways: a conceptual framework to support ecotoxicology research and risk assessment. Environ. Toxicol. Chem. 29, 730–741. 10.1002/etc.34 20821501

[B3] AOP (2024). AOP wiki. Available at: https://aopwiki.org/AOPWiki.

[B4] ASPIS (2023). ASPIS cluster website. Available at: https://aspis-cluster.eu/ASPISclusterwebsite.

[B5] Bhalla D. Steijaert M. N. Poppelaars E. S. Teunis M. Voet M. van der Corradi M. (2023). DARTpaths, an *in silico* platform to investigate molecular mechanisms of compounds. Bioinforma. Oxf. Engl. 39, btac767. 10.1093/bioinformatics/btac767 PMC9825785 36477801

[B6] Bran A. M. Cox S. Schilter O. Baldassari C. White A. D. Schwaller P. (2023). ChemCrow: augmenting large-language models with chemistry tools. Available at: http://arxiv.org/abs/2304.05376 (Accessed December 14, 2023). 10.1038/s42256-024-00832-8 PMC11116106 38799228

[B7] Bus J. S. (2017). “The dose makes the poison”: key implications for mode of action (mechanistic) research in a 21st century toxicology paradigm. Curr. Opin. Toxicol. 3, 87–91. 10.1016/j.cotox.2017.06.013

[B8] Corradi M. P. F. Haan A. M. de Staumont B. Piersma A. H. Geris L. Pieters R. H. H. (2022). Natural language processing in toxicology: delineating adverse outcome pathways and guiding the application of new approach methodologies. Biomaterials Biosyst. 7, 100061. 10.1016/j.bbiosy.2022.100061 PMC9934466 36824484

[B9] Cui L. Yoon S. Schinazi R. F. Sommadossi J. P. (1995). Cellular and molecular events leading to mitochondrial toxicity of 1-(2-deoxy-2-fluoro-1-beta-D-arabinofuranosyl)-5-iodouracil in human liver cells. J. Clin. Investigation 95, 555–563. 10.1172/JCI117698 PMC295512 7860738

[B10] Davis A. P. Wiegers T. C. Johnson R. J. Sciaky D. Wiegers J. Mattingly C. J. (2023). Comparative toxicogenomics database (CTD): update 2023. Nucleic Acids Res. 51, D1257–D1262. 10.1093/nar/gkac833 36169237 PMC9825590

[B11] Fernandes M. R. Pedroso A. R. (2017). Animal experimentation: a look into ethics, welfare and alternative methods. Rev. Da Assoc. Medica Bras. (1992) 63, 923–928. 10.1590/1806-9282.63.11.923 29451652

[B12] GitHub repository (2023). Ontox-project/en-tox en-tox github repository. Available at: https://github.com/ontox-project/en-tox (Accessed December 1, 2023).

[B13] Guan L. Guo L. Zhang H. Liu H. Zhou W. Zhai Y. (2023). Naringin protects against non-alcoholic fatty liver disease by promoting autophagic flux and lipophagy. Mol. Nutr. Food Res. 68, e2200812. 10.1002/mnfr.202200812 38054638

[B14] Hartung T. Vliet E. van Jaworska J. Bonilla L. Skinner N. Thomas R. (2012). Systems toxicology. ALTEX - Altern. animal Exp. 29, 119–128. 10.14573/altex.2012.2.119 22562485

[B15] Honnibal M. Montani I. (2017). spaCy 2: Natural language understanding with Bloom embeddings, convolutional neural networks and incremental parsing.

[B16] Huguet Cabot P.-L. Navigli R. (2021). “REBEL: relation extraction by end-to-end language generation,” in Findings of the association for computational linguistics: emnlp 2021 (Punta Cana, Dominican Republic: Association for Computational Linguistics), 2370–2381. 10.18653/v1/2021.findings-emnlp.204

[B17] Jaylet T. Coustillet T. Jornod F. Margaritte-Jeannin P. Audouze K. (2023). AOP-helpFinder 2.0: integration of an event-event searches module. Environ. Int. 177, 108017. 10.1016/j.envint.2023.108017 37295163

[B18] Ji Z. Lee N. Frieske R. Yu T. Su D. Xu Y. (2023). Survey of hallucination in Natural Language generation. ACM Comput. Surv. 55 (248), 1–38. 38. 10.1145/3571730

[B19] Jin R. McConnell R. Catherine C. Xu S. Walker D. I. Stratakis N. (2020). Perfluoroalkyl substances and severity of nonalcoholic fatty liver in Children: an untargeted metabolomics approach. Environ. Int. 134, 105220. 10.1016/j.envint.2019.105220 31744629 PMC6944061

[B20] Katritsis N. M. Liu A. Youssef G. Rathee S. MacMahon M. Hwang W. (2022). Dialogi: utilising NLP with chemical and disease similarities to drive the identification of Drug-Induced Liver Injury literature. Front. Genet. 13, 894209. 10.3389/fgene.2022.894209 36017500 PMC9395939

[B21] Ku T. Zhou M. Hou Y. Xie Y. Li G. Sang N. (2021). Tebuconazole induces liver injury coupled with ROS-mediated hepatic metabolism disorder. Ecotoxicol. Environ. Saf. 220, 112309. 10.1016/j.ecoenv.2021.112309 34015629

[B22] Lála J. O’Donoghue O. Shtedritski A. Cox S. Rodriques S. G. White A. D. (2023). PaperQA: retrieval-augmented generative agent for scientific research. Available at: https://arxiv.org/abs/2312.07559.

[B23] Maertens A. Golden E. Luechtefeld T. H. Hoffmann S. Tsaioun K. Hartung T. (2022). Probabilistic risk assessment - the keystone for the future of toxicology. ALTEX 39, 3–29. 10.14573/altex.2201081 35034131 PMC8906258

[B24] Mendez D. Gaulton A. Bento A. P. Chambers J. De Veij M. Félix E. (2019). ChEMBL: towards direct deposition of bioassay data. Nucleic Acids Res. 47, D930–D940. 10.1093/nar/gky1075 30398643 PMC6323927

[B25] Monserrate S. G. (2022). “The cloud is material: on the environmental impacts of computation and data storage,” in MIT case studies in social and ethical responsibilities of computing. 10.21428/2c646de5.031d4553

[B26] Neumann M. King D. Beltagy I. Ammar W. (2019). “ScispaCy: fast and robust models for biomedical Natural Language Processing,” in Proceedings of the 18th BioNLP Workshop and Shared Task, Florence, Italy, August 1, 2019, 319–327.

[B27] Van Norman G. A. (2019). Limitations of animal studies for predicting toxicity in clinical trials. JACC Basic Transl. Sci. 4, 845–854. 10.1016/j.jacbts.2019.10.008 31998852 PMC6978558

[B28] Villeneuve D. L. Crump D. Garcia-Reyero N. Hecker M. Hutchinson T. H. LaLone C. A. (2014). Adverse outcome pathway (AOP) development I: strategies and principles. Toxicol. Sci. Official J. Soc. Toxicol. 142, 312–320. 10.1093/toxsci/kfu199 PMC4318923 25466378

[B29] Vinken M. Benfenati E. Busquet F. Castell J. Clevert D.-A. Kok T. M. de (2021). Safer chemicals using less animals: kick-off of the European ONTOX project. Toxicology 458, 152846. 10.1016/j.tox.2021.152846 34216698

[B30] Wang D. Yan J. Teng M. Yan S. Zhou Z. Zhu W. (2018). *In utero* and lactational exposure to BDE-47 promotes obesity development in mouse offspring fed a high-fat diet: impaired lipid metabolism and intestinal dysbiosis. Archives Toxicol. 92, 1847–1860. 10.1007/s00204-018-2177-0 29523931

[B31] Waters M. D. Fostel J. M. (2004). Toxicogenomics and systems toxicology: aims and prospects. Nat. Rev. Genet. 5, 936–948. 10.1038/nrg1493 15573125

[B32] Westergaard D. Stærfeldt H.-H. Tønsberg C. Jensen L. J. Brunak S. (2018). A comprehensive and quantitative comparison of text-mining in 15 million full-text articles versus their corresponding abstracts. PLOS Comput. Biol. 14, e1005962. 10.1371/journal.pcbi.1005962 29447159 PMC5831415

[B33] Yu H. Zhang X. Liu R. Li H. Xiao X. Zhou Y. (2017). Mcl-1 suppresses abasic site repair following bile acid-induced hepatic cellular DNA damage. Tumour Biol. J. Int. Soc. Oncodevelopmental Biol. Med. 39, 1010428317712102. 10.1177/1010428317712102 28681695

[B34] Zaslavsky L. Cheng T. Gindulyte A. He S. Kim S. Li Q. (2021). Discovering and summarizing relationships between chemicals, genes, proteins, and diseases in PubChem. Front. Res. Metrics Anal. 6, 689059. 10.3389/frma.2021.689059 PMC8311438 34322655

